# The Calcium/Calmodulin-Dependent Kinases II and IV as Therapeutic Targets in Neurodegenerative and Neuropsychiatric Disorders

**DOI:** 10.3390/ijms22094307

**Published:** 2021-04-21

**Authors:** Kinga Sałaciak, Aleksandra Koszałka, Elżbieta Żmudzka, Karolina Pytka

**Affiliations:** 1 Department of Pharmacodynamics, Faculty of Pharmacy, Jagiellonian University Medical College, Medyczna 9, 30-688 Krakow, Poland; kinga.salaciak@doctoral.uj.edu.pl (K.S.); aleksandradkoszalka@gmail.com (A.K.); 2 Department of Social Pharmacy, Faculty of Pharmacy, Jagiellonian University Medical College, Medyczna 9, 30-688 Kraków, Poland; ela.zmudzka@uj.edu.pl

**Keywords:** CaMKII, CaMKIV, memory, depression, anxiety, animal studies

## Abstract

CaMKII and CaMKIV are calcium/calmodulin-dependent kinases playing a rudimentary role in many regulatory processes in the organism. These kinases attract increasing interest due to their involvement primarily in memory and plasticity and various cellular functions. Although CaMKII and CaMKIV are mostly recognized as the important cogs in a memory machine, little is known about their effect on mood and role in neuropsychiatric diseases etiology. Here, we aimed to review the structure and functions of CaMKII and CaMKIV, as well as how these kinases modulate the animals’ behavior to promote antidepressant-like, anxiolytic-like, and procognitive effects. The review will help in the understanding of the roles of the above kinases in the selected neurodegenerative and neuropsychiatric disorders, and this knowledge can be used in future drug design.

## 1. Introduction

**Figure 1:**
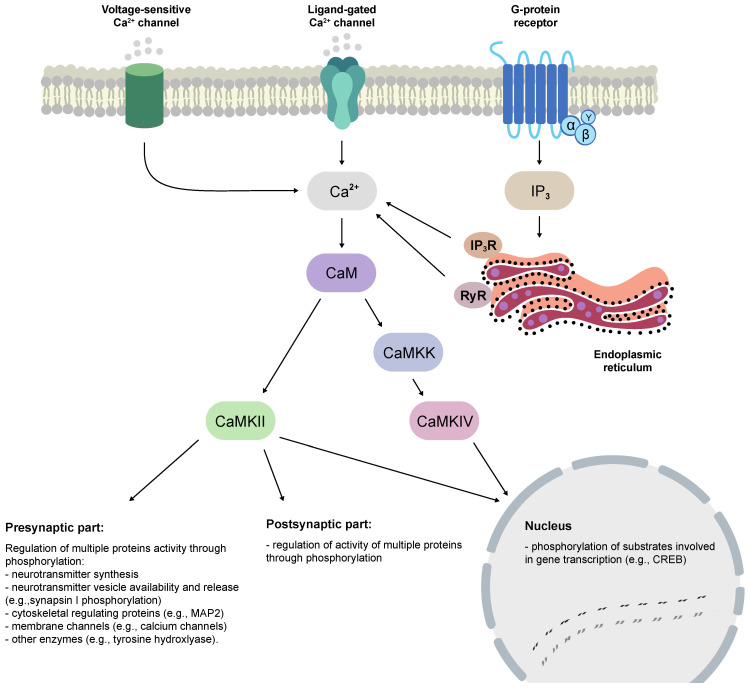
The calcium/calmodulin-dependent kinase (CaMK) II and CaMKIV activation cascades and function in the central nervous system (based on [2,3,4]). Cytoplasmic Ca^2+^ concentrations rise in response to either the activation of voltage-sensitive Ca^2+^-channels, ligand-gated ionotropic channels, or G-protein-coupled receptors in the plasma membrane or by its release from endoplasmic reticulum via IP_3_R or RyR. Next, CaM binds Ca^2+^ ions and either activates CaMKII directly or activates CaMKK, which in turn phosphorylates CaMKIV. CaM—calmodulin, CaMKK—CaMK kinase, CREB—cAMP response element-binding protein, IP_3_—inositol trisphosphate, IP_3_R—IP_3_ receptor, MAP2—microtubule-associated protein 2, RyR—ryanodine receptors.

Calcium (Ca^2+^) controls virtually all aspects of cell life [1]. Cytoplasmic Ca^2+^ concentrations rise in response to either the activation of Ca^2+^-channels in the plasma membrane or by its release from intracellular stores (endoplasmic reticulum, ER). However, to fulfill its role, the Ca^2+^ signal has to be suitably processed. The process is performed by sensor proteins, such as calmodulin (CaM), which can specifically complex Ca^2+^. CaM is a major Ca^2+^-binding protein in the central nervous system. The molecule has four similar domains, each containing one Ca^2+^-binding site. After binding to CaM, Ca^2+^ alters its conformation, and the resulting CaM–Ca^2+^ complex can interact with target proteins and modulate their activity. The major CaM target proteins are Ca^2+^/CaM-dependent kinases (CaMKs), which contribute to many neuronal regulatory pathways, from synaptic plasticity to gene transcription (Figure 1).

CaMKs are a large family of protein kinases that act by Ca^2+^/calmodulin binding and subsequent protein phosphorylation. There are four enzymatically active multifunctional members of CaMKs: CaMKI, CaMKII, CaMKK, and CaMKIV, whose activation affects many downstream targets [5]. Moreover, there are also substrate-specific CaMKs, such as CaMKIII (identified as eukaryotic elongation factor-2 (eEF2) kinase) with one signaling pathway according to the cell or tissue, where they are expressed [6]. Calmodulin kinases play a crucial role in brain functioning, including synaptic plasticity, neuronal transmission, circuit development, and cognition. Among these kinases, CaMKII and CaMKIV are currently the most investigated for neuronal functioning [7] (Table 1). Both CaMKII and CaMKIV belong to the serine/threonine protein kinase family and are regulated by the Ca^2+^/CaM complex. In general, they are arranged into small and large lobe, creating an ATP-binding site and a hinge polypeptide [8]. Although closely related, multifunctional CaMKII and CaMKIV appear to show different structures and models of regulating the autoinhibitory mechanism associated with the activation [9].

This review aimed to systematize the current knowledge from preclinical studies on the role of CaMKII and CaMKIV in the antidepressant- and anxiolytic-like effects, as well as memory formation and its impairment. The review helps in the understanding of the roles of the above kinases in the selected neurodegenerative and neuropsychiatric disorders and use this knowledge in future drug design. 

## 2. CaMKII

**Figure 2:**
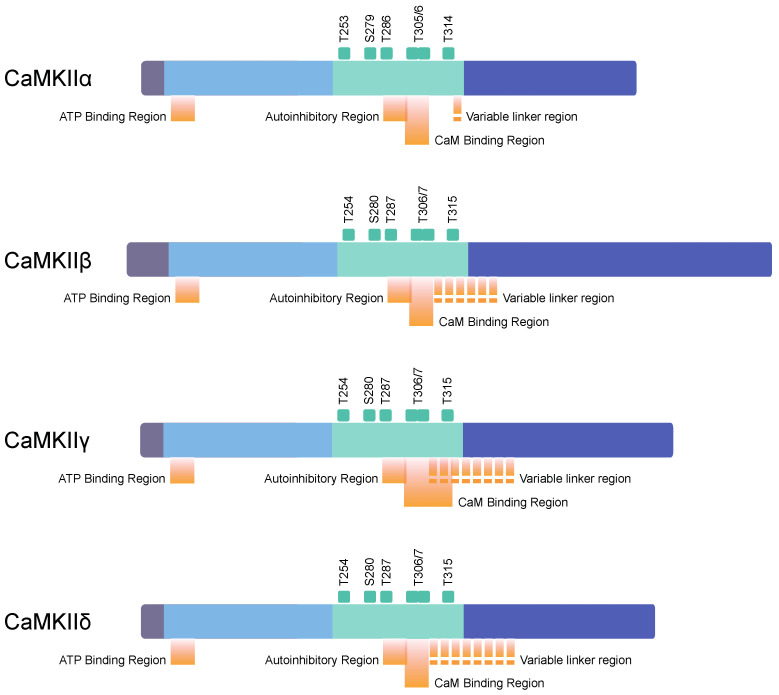
A domain structure of calcium/calmodulin-dependent kinase (CaMK) II (adapted from ref. [1]). We distinguish four CaMKII isoforms: α, β, γ, and δ. CaMKII structure consists of the N-terminal domain (dark gray), catalytic domain (blue), regulatory domain (green), and C-terminal association domain (dark blue). Green squares indicate phosphorylation sites.

CaMKII is found to have four subunit isoforms: α, β, γ, and δ (Figure 2) [44]. Their molecular weights are 50 kDa (α), 60 kDa (β), 59kDa (γ), and 60 kDa (δ), and the number of amino acids are 478, 542, 527, and 533, respectively [4,7,8]. While isoforms γ and δ can be found in every examined tissue, including the brain, α and β are exclusively neuronal, occurring only in the brain, and thus these are of main interest in this paper [20,45].

### 2.1. Genetics

Amino acid sequences of subunits are homologous at least at the 84% rate, but they are products of four different genes [45]. The corresponding kinase isoforms isolated from mammalian tissues are highly conserved, e.g., the coding region of mRNA in rats is 93% consistent with the human mRNA sequence. Moreover, human and rat α isoforms at the protein level are identical in amino acid composition [46].

The encoding gene and exon–intron organization of α isoform were analyzed. The transcription initiation site was placed at −149 or −147 bases from the first ATG. The cDNA coding region consists of 18 exons spanning over 50 kilobase pairs. Each of the functional units, such as calmodulin-binding site or ATP-binding site, is encoded by a single exon [47,48,49].

The genomic organization of the β isoform was also demonstrated. The transcription initiation site was at G, −78 from the first ATG. Gene consisting of 21 exons spans more than 80 kbp, creating the 20-exon coding sequence. The exon/intron junction structure is completely conserved between α and β genes except for the β-specific insertion and near the stop codon. In contrast, the size of introns is very different between these two genes [50,51].

### 2.2. Structure of Subunits

Each subunit consists of the N-terminal kinase domain, CaM-binding regulatory domain, and C-terminal association domain responsible for the existence of holoenzymes. Subunit δ has an additional segment of 21 amino acid residues at the C-terminus [8,19].

Kinase domain, also named catalytic domain (residues 1-278, numbering for rat α isoform), contains ATP- and substrate-binding site. The reaction of substrate phosphorylation runs within this domain [52].

Regulatory or autoinhibitory domain (residues 273-317; 290-314 is Ca^2+^/CaM-binding site) in inactivated state interacts with the catalytic domain, limiting access to the ATP-binding site, and also prevents Ca^2+^/CaM-independent phosphorylation. Although it was crystallized in helical conformation, the autoinhibitory domain in the basal state forms α helix only in the N-terminal part. When activated by Ca^2+^/CaM, it changes conformation enabling the activity of the kinase. This domain contains Thr268, the primary site of autophosphorylation [8,53,54]. 

Association domain (residues 345-472) forms the hub complex of holoenzyme (described in the following section). It acts as a flexible linker region that facilitates the formation of a variety of conformational states, which usually incompletely match the average reconstruction model (including asymmetric forms) [55].

### 2.3. Structure of the Holoenzyme

From the day of the first discovery of CaMKII, efforts were made to establish the three-dimensional structure of its holoenzyme. Various arrangements were suggested, e.g., two parallel hexametric rings [52,53,56,57]. Recently, in 2017, Myers and colleagues were able to obtain pseudoatomic models of CaMKIIα holoenzyme in an extended, activation-competent conformation. Their work confirmed that CaMKII is organized into a flower-like shape, with the central density of protein surrounded by 8–10 smaller peripheral fragments—‘petals’ [46,55]. This ring is, in fact, an octamer or decamer of kinase subunits. Each one’s association domain is a flexible linker gathering in the central hub complex. Regulatory and catalytic domains form peripheral densities [46]. 

Typical neuronal holoenzyme is built of α and β subunits, 12 in total, which create homo- or heteropolymeric molecules in the various quantitative ratio (depending on investigated tissue). For instance, the majority of forebrain enzyme molecules possess 10 petals of α subunits only, and the cerebellum enzyme mostly consists of eight petals, β subunits only [20,46,58]. 

### 2.4. Mechanism of Activation and Autoregulation

The inactive state of the kinase is guarded by the autoinhibitory domain that is positioned to sterically occlude the substrate-binding site and to disrupt ATP binding. Two neighboring autoinhibited catalytic domains of the holoenzyme create symmetric dimer that is held together by a coiled coil formed by their regulatory domains [53,58]. Holoenzyme structures with 1–3 kinase domain pairs appear to be the most common, while configuration with all kinase domains arranged as dimer appears rare (represented only ≈2.5% of the investigated population) [55]. 

Activation starts when the Ca^2+^/CaM complex attaches to the binding site within the regulatory domain, which changes the conformation of its C-terminus into helical and pushes the N-terminal part into disordered conformation. It causes separation of previously dimeric catalytic domains and starts the intersubunit reaction. Subunit activated by Ca^2+^/CaM acts like a kinase, and adjacent subunit is its substrate. Exposed Thr286 undergoes phosphorylation, and anionic phosphate moiety blocks reformation of the coiled coil and maintains dissociation of a catalytic domain from its regulatory domain. This extended conformational change disables inhibition of ATP binding, enables phosphorylation of various substrates, consolidates affinity of CaM to autophosphorylated kinase (K_off_ is decreased 23,000-fold), and enhances the binding of active kinase to synaptic sites [8,58,59]. After the autophosphorylation, each catalytic domain adopts an active conformation that is stabilized through base–acid interactions between the association domain and linker of the regulatory domain. This interplay prevents reformation of the inhibitory coiled coil, even when levels of Ca^2+^ drop and Ca^2+^/CaM dissociates. Taken together, activation through autophosphorylation of Thr286 is Ca^2+^/CaM-dependent, but it results in partially Ca^2+^/CaM-independent kinase activity by reducing the effectiveness of inhibitory domains [53,54].

The autoregulation cycle ends up in secondary autophosphorylation of Thr305/Thr306 (called capping) occurring Ca^2+^-independently. It blocks CaM rebinding and favors dissociation of CaMKII from protein anchors in the synaptic site [8,46].

### 2.5. CaM Trapping

Autophosphorylation is supposed to promote high-affinity CaM binding (called trapping) by enabling favorable interactions between Glu120 and Met124 of CaM and residues 293-298 of the kinase. When CaMKII is unphosphorylated, Phe293 and Asn294 have strong connections to Phe98 and Ile205 from the catalytic site. Thus, Phe293 and Asn294 either stabilize the autoinhibited form of the enzyme or, when Thr286 is phosphorylated, consolidates the CaM-bound form. CaM trapping prolongs kinase activity and prevents immediate autophosphorylation of Thr305/Thr306. It is also likely the ground for the ability of CaMKII to decode the frequency of Ca^2+^ oscillations into distinct levels of enzyme activity in vitro and may play a similar role in vivo [58].

### 2.6. Other Regulatory Factors

As proved in previous sections, the activity of CaMKII is closely linked to calmodulin and Ca^2+^. In general, if the concentration of Ca^2+^ is high, Thr286 autophosphorylation spreads rapidly through subunits of the holoenzyme, leading to the onset of Ca^2+^/CaM-independent activity. When Ca^2+^ level falls, Thr286 is usually dephosphorylated before activation can proceed [53]. However, CaMKII turned out to be able to decode the number and frequency of Ca^2+^ oscillations. In this respect, the arrangement of the subunits in a holoenzyme is crucial. Appropriate pulsatile activation of CaMKII may extend its activity way beyond the period of Ca^2+^ elevation. This feature may play an important role in frequency-dependent changes in synaptic plasticity [60,61]. 

The autophosphorylation occurs at several other sites of CaMKII, and one of these is Thr253. This exact process is relative to Thr286 autophosphorylation, favored by low concentration of ATP and increases in response to depolarization. It enhances holoenzyme binding to proteins in subcellular fractions enriched in PSDs (post-synaptic densities) but does not affect autonomous activity nor Ca^2+^/CaM-dependent activity [62]. Several posttranslational modifications of the regulatory domain, including oxidation, nitrosylation, and GlcNacylation, may also cause changes in enzyme functioning, especially Ca^2+^/CaM-independent activity [8].

CaMKII targeting multiple anchoring proteins can occur thanks to several distinct interactions. For example, the association domain that has isoform-specific inserts may bind the β-isoform to actin filaments. The Thr286 docking site, exposed by the activation of the kinase, is used to anchor the enzyme to the NMDA receptor subunit NR2B. This way, interactions between domains of CaMKII and multiple anchoring proteins may lead to clustering of holoenzymes to a particular site, such as the synapse [63]. 

### 2.7. Function

CaMKII is expressed throughout the body, phosphorylates a wide range of substrates, and regulates numerous cellular functions. CaMKII regulates cardiac functioning (for a review, see [26,27,28]), hepatic glucose production and insulin signaling [64,65], vascular smooth muscle cell function [66], cell cycle progression and fertility [67,68,69], and pain [29,70], as well as the immune system [23,24,25]. Moreover, it plays a critical role in the proliferation, differentiation, and survival of various cancer cells [71,72,73,74,75]. Moreover, the aberrant phosphorylation of CaMKII is the cause of neurological symptoms of Angelman syndrome [39]. However, since CaMKII is expressed most abundantly in neurons, it regulates many aspects of neuronal function, including neurotransmitter synthesis and release, modulation of ion channel activity, cellular transport, cellular morphology, and neurite extension [76,77,78,79]. 

CaMKII controls various neurotransmitters systems; however, the presynaptic CaMKII may act as either enhancer or inhibitor of neurotransmitter release depending on properties of specific synapses [76,80,81,82]. Moreover, CaMKII inhibition induces dysregulation of neuronal calcium and glutamate homeostasis resulting in increased excitability and subsequent apoptosis [83]. The blockade of CaMKII leads to an increase in the extracellular glutamate level. CaMKII influences the numerous ion channels in the organisms. Studies showed that this kinase regulates the conductivity of the various types of sodium, potassium, and calcium channels [84]. Considering the neuronal cells, CaMKII enhances voltage-gated sodium channel Nav1.6 activity and neuronal excitability [79]. Moreover, by modulating the voltage-gated calcium (CaV)2.1 channels that conduct P/Q type Ca^2+^ currents, CaMKII regulates the neurotransmitter release [85]. 

During neuronal development, the proper CaMKIIβ functioning is required for the correct neuronal migration [86]. This isoform controls the dendrite growth and arborization during the developmental period, and the lack of CaMKIIβ resulted in the longer dendrites [87]. Moreover, the inhibition of CaMKII impairs axon growth in both the peripheral and central nervous systems via affecting the length of the F-actin [88]. More specifically, it is the isoform β that promotes the synapse and spine formation and elongation via its F-actin bundling activity [89,90]. Moreover, these kinases take part in the regulation of neurite extension. Interestingly, the isoforms of CaMKII play a different role in neuronal plasticity—CaMKIIα predominantly regulates the synaptic strength whilst CaMKIIβ controls dendritic morphology, neurite extension, and synapse number [89]. Moreover, CaMKII plays an essential role in long-term plasticity, learning, and memory consolidation (detailed description below) [91,92,93]. 

## 3. CaMKIV

**Figure 3:**
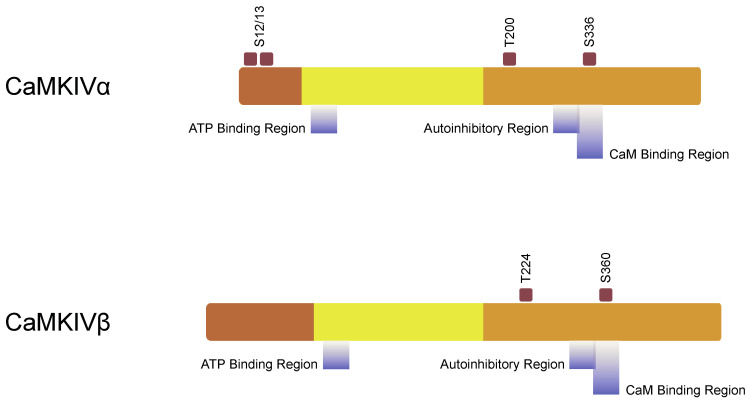
A domain structure of calcium/calmodulin-dependent kinase (CaMK) IV (adapted from ref. [1]). We distinguish two CaMKIV isoforms: α and β. CaMKIV structure consists of the N-terminal domain (dark brown), catalytic domain (yellow), regulatory domain (brown). Brown squares indicate phosphorylation sites.

The CaMKIV was first described as ‘CaM-kinase GR’ because of its abundance in the cerebellum’s granular cells [21]. There are two isoforms, CaMKIVα and CaMKIVβ (Figure 3), and are both expressed as monomers with the molecular weights of 65 kDa (α) or 67kDa (β) [94,95]. A mature polypeptide of CaMKIV consists of 469-473 amino acids [9,96]. CaMKIV is not as ubiquitous among tissues as CaMKII is; its expression is restricted to nervous tissue, thymus (especially T-lymphocytes), spleen, and testis [22]. 

### 3.1. Genetics

The gene of 42 kbp encoding the enzyme consists of 12 exons and 11 introns and is considered as encoding both α and β forms of kinase as well as calspermin—a testis-specific calmodulin-binding protein. These are all produced by alternative sites of transcription initiation. CaMKIVβ comes from the longest transcript. The promoter of CaMKIVα is located within the first intron, and calspermin has its promoter and first exon located in intron 10 [30]. When this is considered, it is not surprising that the sequence of the C-terminal 169 amino acids of the kinase is identical to that of calspermin [29].

### 3.2. Structure of Subunit

The subunit is comprised of the protein kinase domain (residues 42–296), autoinhibitory domain (297–336), overlapping PP2A-binding domain (302–319), and CaM-binding domain (318–337). The most significant site of phosphorylation is Thr196 (numbering refers to rat isoform) [97]. 

The N-terminal part of the human CaMKIV is highly conserved compared with rat and mouse parts. In contrast, the C-terminal part is very less conserved (≈37% sequence identity) with rat and mouse CaMKIV. These kinases possess a parallel sequence motif, X-Arg-X-X-Ser/Thr, with X meaning a hydrophobic residue. Altogether, it creates an essential site for phosphorylation [98]. 

The first 291 amino acids of CaMKIV have been found to be 58% homologous to the first 314 amino acids of CaMKIIα (if conservative amino acid substitutions are considered). The entire amino acid sequences are 42% homologous [94].

Within the large lobe of the kinase domain, an activation loop with Thr196 is located. This site needs to be phosphorylated for proper folding of the ATP-binding pocket and optimal activity of kinase [8].

### 3.3. Mechanism of Activation and Autoregulation

In the resting state autoinhibitory sequence interacts with the catalytic domain. This inhibition is not competitive with ATP but with the peptide substrate [9,95]. Ca^2+^/CaM binding to the regulatory segment enables Thr196 phosphorylation by CaMKK. It results in conformational changes that abolish the interaction between the catalytic core and autoinhibitory domain and generation of highly active form of CaMKIV, partially Ca^2+^/CaM-independent. The active site becomes fully exposed for substrate binding and its phosphorylation. The third level of activation concerns autophosphorylation of serine residues at the N-terminal part of CaMKIV, Ser11, and Ser12. 

When the Ca^2+^ level decreases significantly, it is followed by the dissociation of Ca^2+^/CaM and PP2A binding, which causes dephosphorylation of CaMKIV and its deactivation [95,98,99,100].

### 3.4. Other Regulatory Factors

CaMKIV undergoes several posttranslational modifications, one of which is GlcNacylation. The addition of O-GlcNac to Ser/Thr residues participates in regulating the activation and function of CaMKIV [101]. 

One of the factors responsible for targeting CaMKIV may be polyglutamate sequences present within the C-terminal part of the kinase. They are supposed to target the enzyme to the nuclear chromatin of specific neurons [94].

### 3.5. Function

CAMKIV also controls various biological activities starting with the regulation of the activity of the numerous transcription factors such as cAMP-response element binding protein (CREB), myocyte enhancer factor 2D, JUN, ATF-1, SRF, and retinoic acid-related orphan receptor-alpha [65,66,67,68]. CaMKIV controls blood pressure [34], inflammatory responses, T cell maturation [35,36,37], bone growth and metabolism [102,103], microtubule dynamics [38], cell cycle and apoptosis [104,105,106], and development of neurons and male germ cells [105,107,108]. CaMKIV is required for activity-dependent dendrite elaboration: branching and elongation [109]. CaMKIV also contributes to the dendritic and neurite growth via TRPC6 channels, which activate CaMKIV/CREB pathway [110,111]. Overexpression and increased activity of this enzyme are linked with various types of cancer such as hepatocellular carcinoma, breast cancer, neuroblastoma, prostate cancer, and AML (for a review, see [42]). Moreover, its function in the hippocampus contributes to memory consolidation and long-term potentiation (detailed description below) [112,113].

## 4. Role in Neurodegenerative and Neuropsychiatric Disorders

**Figure 4:**
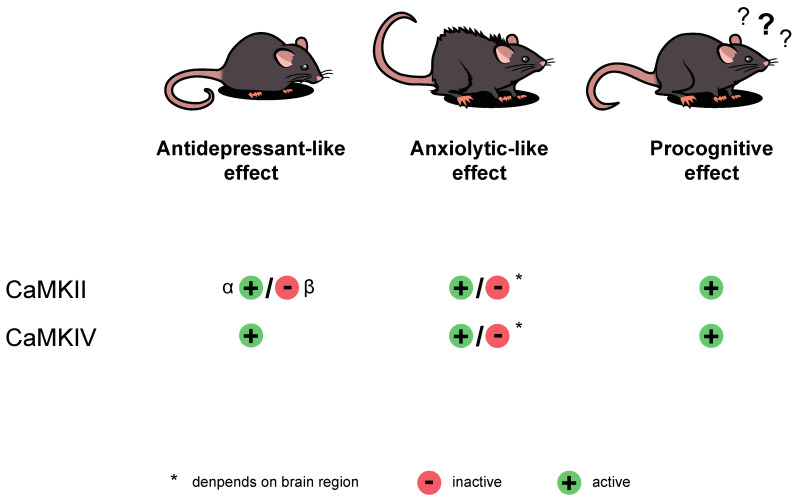
The role of calcium/calmodulin-dependent kinase (CaMK) II and IV in antidepressant-like, anxiolytic-like, and procognitive effects. The procognitive effect requires activation of both CaMKII and CaMKIV. For antidepressant-like activity to be induced, CaMKIIα and CaMKIV need to be activated, and CaMKIIβ inactivated. The data on the anxiolytic-like effect depend on the studied brain region.

Since both CaMKII and CaMKIV are widely distributed across the nervous system, they participate in many crucial neuronal processes. Their abnormal functioning induces negative changes and consequently may lead to the diseases’ development. Here, we discuss the role of CaMKII and CaMKIV in depression and anxiety (Figure 4), whose prevalence is increased, especially since the emergence of the recent coronavirus disease 2019 (COVID-19) pandemic. Moreover, since both kinases have a prominent role in learning and memory, we focus not only on the cognitive deficits but also on how important the proper activity of these enzymes is for memory functioning (Figure 4).

## 5. Depression-Like State in Rodents

Depression is a devastating mental disorder due to abnormal changes in different brain areas such as the hippocampus or lateral habenula [114]. The expression and level of CaMKII and CaMKIV in animals’ hippocampi are also commonly altered in depression [115]. Here, we summarize the most important preclinical data on the role of CaMKII and CaMKIV in the pathophysiology of depression and antidepressant drug action.

### 5.1. CaMKII

CaMKII is the kinase involved in the pathophysiology as well as in the antidepressant activity of drugs. CaMKIIα knockout mice did not show any changes in behavior during the forced swim test, a test used to evaluate antidepressant-like effects in rodents [116]. Moreover, neither chemogenetic activation nor inhibition of CaMKIIα-positive forebrain excitatory neurons altered the animals’ behavior in the forced swim and tail suspension tests, both detecting depressive-like states in animals [117]. However, Yamasaki and colleagues have shown that heterozygous CaMKIIα knockout mice presented depressive-like behavior in the forced swim test [118]. Such discrepancies may result from different strategies used to block the CaMKII activity. On the other hand, the overexpression of CaMKIIβ in the lateral habenula or in the CA1 region of the hippocampus produced behavioral effects such as the increase in the immobility time as well as the reduction in the preference for the sucrose solution [15,119]. Moreover, the reversal of the depressive-like phenotype of depressed rats occurred after CaMKIIβ deletion in the lateral habenula or hippocampus [15,119]. 

Various animal models of depression induce changes CaMKIIβ expression in several brain structures. CaMKIIβ level was increased in congenitally learned helpless rats, after the acute learned helplessness, chronic mild stress, or lipopolysaccharide injections [15,119]. Moreover, thyroidectomy rats displayed the depressive-like phenotype in the forced swim test with the elevated CaMKIIβ expression in the lateral habenula [16]. The elevated signaling of CaMKIIβ induced by stress was most likely connected with a depression-like state because of the impact on the formation and retention of aversive memories [114]. However, the olfactory bulbectomized rats showed a decrease in the level of CaMKIIα in the hippocampus [120,121]. Moreover, the unpredictable chronic mild stress procedure diminished the CaMKIIα level in mice [122]. It seems that CaMKII isoforms may play opposite roles in the development of depressive-like phenotype. On the other hand, one study indicated that acute but not chronic stress was responsible for increasing both isoforms of CaMKII [123,124]. Interestingly, in one of the postpartum depression models in rats, the expression of CaMKIIα, but not CaMKIIβ, was decreased [125]. Moreover, the inhibition of CaMKII activity by the intra-lateral habenula infusion of KN-62, a CaMKII inhibitor, resulted in decreased depressive-like behavior and alcohol intake in rats [126].

The CaMKII is a kinase significantly implicated in the mechanism of antidepressant drugs’ action. The common finding is that long-term treatment with antidepressants is connected with adaptive and plastic changes in molecular and cellular neuronal signaling [127]. In the social defeat model of depression, the downregulation of CaMKIIα was necessary to obtain the antidepressant-like effect of selective serotonin reuptake inhibitors (SSRIs) [128]. On the other hand, studies confirmed the upregulation of CaMKII following chronic antidepressant treatment and further neuronal changes, resulting in modulation of synaptic transmission [129,130]. Moreover, the long-term treatment with other SSRIs such as fluvoxamine or paroxetine as well as venlafaxine, which is a serotonin and noradrenaline reuptake inhibitor, resulted in increased autophosphorylation and activation of CaMKII in the hippocampus [131,132]. Repeated administration of imipramine led to the downregulation of CaMKIIβ in the lateral habenula of congenitally helpless rats [15]. Reduction of CaMKIIβ in the CA1 by antidepressant treatment with fluoxetine alleviated depression symptoms [119]. Chronic treatment with fluvoxamine and desipramine increased the activity of presynaptic CaMKII in rat’s frontal and prefrontal cortex [10], whereas long-term administration of desmethylimipramine or S-adenosylometionine resulted in increased activity of CaMKII in the hippocampal synaptic vesicles [133]. Repeated administration of imipramine decreased the activity and level of CaMKII in the hippocampal soluble fraction, whereas acute and repeated imipramine treatment increased the activity of CaMKII in the particulate fraction [134]. The antidepressant treatment increased activity of CaMKII in the particulate fraction that was probably due to the translocation of the kinase from the soluble compartment.

Some researchers indicated that chronic administration of fluoxetine may cause the epigenetic control of CaMKIIα promoter in nucleus accumbens, influencing the transcription factor ΔFosB binding in animals [128].

Moreover, CaMKII is essential for the antidepressant-like action of ketamine. CaMKII inhibitor prevented the ketamine-induced decrease in immobility in the forced swim test in mice [135]. Similarly, in the mouse model of depression induced by lipopolysaccharide, the extrasynaptic increase in CaMKIIα expression was attenuated by ketamine administration and treatment with CaMKIIα inhibitor KN-93 [136]. These effects were connected with the downregulation of GluN2B receptor localization and phosphorylation in mouse hippocampal neurons.

On the other hand, the phosphorylation of CaMKII in the hippocampal region CA1 of olfactory bulbectomized mice was increased after repeated administration of sunifiram, a novel pyrrolidone nootropic drug, but the treatment did not ameliorate depressive-like behavior in the tail suspension test in animals [120]. Interestingly, chronic nefiracetam treatment, another prototype memory enhancer, resulted in elevated CaMKII activity and improved depressive-like behaviors in mice [137]. Such activation of CaMKs and antidepressant-like effect might be a result of CREB elevated phosphorylation in the amygdala and prefrontal cortex. The T-type calcium channel enhancer SAK3 reversed the reduction in CaMKII level in olfactory bulbectomized rats and showed the antidepressant-like effects [138]. 

There are also studies with Guanxin Danshen formula, which is a natural botanical drug composition with potential neuroprotective and antidepressant-like activity. Xie and colleagues confirmed the antidepressant-like effect of Guanxin Danshen formula in rats exposed to the unpredictable chronic mild stress [139]. They observed reduced immobility time in the forced swim test and the tail suspension test as well as increased sucrose consumption in animals with a concomitant decrease in the phosphorylation of intracellular CaMKII. These results might suggest that the antidepressant-like effects of the tested Guanxin Danshen formula are related to changes in the CaMKII pathway. The citrus flavonoid 3,5,6,7,8,3′,4′-heptamethoxyflavone reversed the corticosterone-induced changes, both behavioral and molecular [140]. Flavonoid restored the decrease in the phosphorylation of CaMKII caused by chronic glucocorticoid administration [140]. Studies using ursolic acid, creatine, ferulic acid, or (octylseleno)-xylofuranoside showed that the reduction of immobility time in the tail suspension test in mice might be reversed by administration of KN-62 (a CaMKII inhibitor) [141,142,143,144,145]. Similarly, the antidepressant-like effect of memantine and 7-fluoro-1,3-diphenylisoquinoline-1-amine in forced swim test in mice was blocked by KN-62 [146,147]. It suggests that ursolid acid and creatine, as well as ferulic acid, (octylseleno)-xylofuranoside, memantine, and 7-fluoro-1,3-diphenylisoquinoline-1-amine antidepressant-like activities might be associated not only with monoaminergic system modulation but also with the activation of CaMKII [144,148,149]. Another natural compound, baicalin, which is a flavonoid glycoside, was also tested in a mice model of chronic unpredictable stress. Results indicated its neuroprotective and antidepressant activity in the sucrose preference test, the tail suspension test, and the forced swim test in mice [150]. The administration of baicalin also reduced the upregulated level of CaMKII, suggesting that its antidepressant-like effect might be due to CaMK pathway regulation.

Animal studies also suggested that antidepressant-like effects of deep brain stimulation depend on CaMKII phosphorylation in lateral habenula as well as its downregulation in the infralimbic cortex [151]. Moreover, electroconvulsive therapy downregulated the expression and level of CaMKIIα in rat hippocampus in an animal model of depression [152]. Furthermore, the electroconvulsive shock treatment affects the CaMKII activity in the soluble and particulate fraction of the hippocampus [134].

Taken together, we can generalize that different isoforms of CaMKII play different roles in depressive-like phenotype and antidepressant response. The increase in CaMKIIβ and decrease in CaMKIIα are linked with depressive-like phenotype in animals (Table 2). Moreover, most studies suggest that chronic antidepressant treatment is directly associated with changes in CaMKII activity. The majority of antidepressants increase the expression of CaMKIIα and decrease the expression of CaMKIIβ. Presented studies provide new perspectives in the drug design process, as compounds modulating CaMKII, particularly increasing the activity of CaMKIIα and decreasing CaMKIIβ, may help treat mood disorders.

### 5.2. CaMKIV

CaMKIV is one of the key regulators of different intracellular processes such as neuroprotection and neuroplasticity [173]. There are only several animal studies describing the involvement of CaMKIV in depression. The kinase plays a crucial role in stress-related behavior in CaMKIV knockout mice [156]. CaMKIV knockout mice showed no changes in behavior in the forced swim test and tail suspension test compared with the wild-type mice [153]. However, other scientists proved that CaMKIV knockout mice displayed a depressive-like phenotype given the increase in the immobility time in the forced swim test and tail suspension test [154].

CaMKIV expression is altered in various animals’ models of depression. Patki and colleagues showed that social defeat stress in rats reduced the level of CaMKIV in the hippocampus, leading to a depressive-like state in animals assessed by the sucrose preference test [17]. Similar results were observed both in the unpredictable chronic mild procedure and in the olfactory bulbectomized rats—depressive-like phenotype contributed to the decrease in the CaMKIV phosphorylation [137,174]. 

A number of drugs, compounds, or therapies elicit their antidepressant-like activity probably via CaMKIV pathway’s modulation. In CaMKIV knockout mice, chronic nicotine treatment decreased depressive-like behavior via alfa7-nAChRs (nicotinic acetylcholine receptors) and subsequent activation of this signaling pathway [43]. The reversal of depressive-like phenotype in CaMKIV knockout mice also occurred after sigma-1 receptor stimulation. That suggests that sigma-1 receptor activation compensates the negative effects caused by CaMKIV depletion, probably due to increased hippocampal neurogenesis [154,175]. In olfactory bulbectomized mice SAK3, the T-type calcium channel enhancer nefiracetam, novel procognitive compound, and memantine reversed the depressive-like behaviors and the reduction in the CaMKIV phosphorylation [137,138,176]. Interestingly, in the rat model of depression induced by unpredictable chronic stress, the electroacupuncture and chronic fluoxetine treatment resulted in hippocampal upregulation of CaMKIV and the alleviation of depressive-like symptoms [174]. Moreover, the CaMKIV is involved in hippocampal cell proliferation with chronic fluoxetine treatment [177]. 

Studies of hyperforin, which is an active constituent of St. John’s wort extract with documented antidepressant activity, indicated that the CaMKIV is one of the signaling pathways that mediates the improvement of synaptic plasticity via TRPC6 (non-selective cationic transient receptor canonical 6) channels activation) and probably explains the pharmacological effects of hyperforin [110].

Presented data confirmed the relation of CaMKIV with the pathophysiology of depression and allowed us to consider CaMKIV as a potential target for novel antidepressant therapies. The CaMKIV deletion is linked to a depressive-like state in animals, and many studies reported the decreased kinase phosphorylation in animal models of depression. Thus, we may conclude that CaMKIV activation is preferable for antidepressant-like activity.

## 6. Anxiety-Like State in Rodents

Anxiety is a mental disorder leading to excessive nervousness, fear, and apprehension, and its prevalence increases worldwide [178]. Given the critical role of CaMKII and CaMKIV in regulating various forms of plasticity as well as in the multiple signaling systems [115,179], there are numerous studies considering how these kinases underlie the pathophysiology and treatment of anxiety disorders. Here, we gathered up-to-date knowledge about the role of CaMKII and CaMKIV in the etiology of anxiety-like behaviors in animals. We also discuss the role of these kinases in the anxiolytic-like responses induced by anxiolytics.

### 6.1. CaMKII

Deletion of genes coding CaMKII isoforms led to specific changes in phenotype, and anxiety-like responses in particular. CaMKIIβ knockout mice exhibited decreased anxiety-related behavior assessed during the elevated plus maze test and the open field test [40]. In both cases, animals spent more time in the open areas. Moreover, the CaMKIIα knockout mice displayed a decrease in the fear-related responses [157]. On the other hand, the overexpression of CaMKIIα in the forebrain resulted in increased anxiety-like behavior in various tests like the open field, elevated zero maze, and light/dark transition [160]. This suggests the kinase expression controls anxiety-like phenotype. However, the pharmacogenetic activation of CaMKIIα in the prefrontal cortex reduced the anxiety-like behavior in the elevated plus maze test, but not in the open field test [11]. Moreover, another group of scientists showed that mice with activated CaMKIIα-positive forebrain excitatory neurons displayed anxiolytic-like behavior in the open field, light–dark avoidance, and the elevated plus maze tests [117]. Moreover, CaMKII signaling importance is also evident when the Thr286 autophosphorylation is eliminated [14]. Mice spent more time in the open arms of the elevated plus maze, and therefore they displayed anxiolytic-like phenotype. Easton and colleagues also reported changes in the behavior of T286A CaMKIIα autophosphorylation-deficient mice [180]. Their behavior was anxiolytic-like in the elevated plus maze test and light/dark test. However, these mice also were hyperactive during these tests, but this hyperlocomotion appeared only when animals were exposed to novel environments that are threatening for them [180]. That suggests the CaMKII may mediate changes in locomotion in a stressful situation instead of anxiety- or anxiolytic-like phenotype per se.

CaMKII role in the regulation of fear-related phenotype is more complex and more profound than we have thought. Exposure of rats to predator odor induced the defensive burying response and the activation of CaMKII-positive subpopulations of the amygdala [181]. Moreover, during drug withdrawal, scientists observed symptoms of increased anxiety and fear in animals, which may be linked to CaMKII-mediated signaling. The expression of total CaMKIIα in postsynaptic density may regulate the glutamatergic plasticity and the flurazepam withdrawal symptoms by complexing with GluN2B NMDARs [182,183]. CaMKII also regulates the anxiety-like responses in the offspring of morphine-addicted parents—these mice show increased anxiety-like behavior and CaMKII inhibition decreases fear in animals [184]. 

Moreover, the phosphorylation of CaMKII can be regulated via various receptors or enzymes. Mice without soluble epoxide hydroxylase exhibit anxiety-like behavior as a result of the hyperphosphorylation of CaMKII and glycogen synthase kinase 3 α/β (GSK3α/β) [185]. Moreover, the decreased level of CaMKII in the striatum was observed in mice without connexin36; the protein presents in gap junctions between interneurons in several brain structures [186]. This was eventually related to behavioral changes such as anxiety in the light/dark box. 

Interestingly, CaMKII plays a crucial role in the 5-HT1A-mediated pathways determining the anxiety-like response during the developmental period [12]. The 5-HT1A knockout mice exhibited anxiety-like behavior during tests, and they showed increases in CaMKIIα level in the hippocampus. However, this change in phosphorylation occurred only at a specific life period, and during that time the CaMKII regulated and formed the anxiety-like phenotype of adult mice [12]. This finding was confirmed by causing a mutation of CaMKIIα that decreases phosphorylation, which abolished the fear-related phenotype [12]. The importance of CaMKII regulation in anxiety by serotonin and serotonin receptors was also investigated in 5,7-dihydroxytryptamine (5,7-DHT)-induced serotonin depleted mice [155]. The study concluded that low serotonin levels were associated with anxiety-like phenotype, and the CaMKII facilitated such behaviors, since the CaMKII knockdown attenuated the fear responses in animals measured in the open field test [155]. 

The CaMKII inhibitors are an attractive therapeutic option. TatCN21 peptide (CaMKII inhibitor) reversed the anxiety-like state in the elevated plus maze test and open field test in rats with global cerebral ischemia [41]. Thus, the inhibition of CaMKIIα over-activation in global cerebral ischemia exerted protective effects on animals’ behavior. Diazepam increased the level of phospho-CaMKII in the hippocampus and striatum [187]. It may have been due to GABAergic-induced increased calcium level or interaction with MEK-ERK pathway. Moreover, memantine, a drug used in memory disorders, reduced the anxiety-like behavior in rats that suffered from alcohol withdrawal, probably via the CaMKII pathway [188]. Genistein, a natural isoflavone, caused anti-anxiety effects in a post-traumatic stress disorder model by enhancing the serotonergic system and subsequent CaMKII/CREB signaling pathway in the amygdala [189].

To sum up, CaMKII participates in mediating the anxiolytic-like response in animals. The depletion of both α and β isoforms causes an anxiolytic-like effect, whereas their overexpression leads to an anxiety-like state (Table 2). Moreover, the pharmacological inhibition of CaMKII is related to a decrease in fear. However, most anxiolytics elevate the level of CaMKII in the various brain structures. This suggests that the balance in the CaMKII levels in different brain structures is important for an anxiolytic-like effect.

### 6.2. CaMKIV

CaMKIV is known for being involved in behavioral alterations, mainly by phosphorylating a significant transcription factor—CREB and CRE modulator (CREM). CaMKIV knockout mice showed a decrease in anxiety-like response in common behavioral paradigms such as elevated plus maze test, dark–light emergence test, light/dark box, and acoustic startle and prepulse inhibition [156]. This suggests the vital role of CaMKIV-mediated signaling is essential for anxiolytic-like behavior, probably due to its influence on CREB and CREM expression. Another possible explanation may be linked to the differences in expression of anxiety- and stress-related genes in CaMKIV knockout mice. For example, a more than twofold decrease in oxytocin mRNA was observed in knockout mice [156]. Since oxytocin controls fear and emotions, the changes in its expression caused by CaMKIV may underpin the behavioral phenotype. The decrease in anxiety-like behavior in mice with depleted CaMKIV was also observed in the elevated plus maze test, light/dark test, and marble-burying tests [153,158]. 

The alternation in CaMKIV level is also seen in animals subjected to stress [17]. Social defeat modifies animal behavior, including fear responses. Depressed rats displayed the anxiety-like behavior with a concomitant decrease in CaMKIV level in the hippocampus. 

On the other hand, the role of CaMKIV in anxiety seems to be region-specific. Mice with dominant-negative CaMKIV variant in nucleus accumbens elicited anxiogenic behavior in the light/dark test [18]. That suggests CaMKIV modulates the anxiety-like response of animals, but the final effect is the result of kinase function in the whole brain. [18]. 

Interestingly, the CaMKIV influences anxiety-like behavior in drug- or substance-dependent animals. The research on nicotine dependence and withdrawal in CaMKIV knockout mice was conducted using a conditioned place preference test [159]. Results showed that in genetically modified mice, nicotine conditioned place preference test was attenuated compared to control. After withdrawal, the anxiety-related behavior was not observed in the knockout group [159]. These findings suggest that CaMKIV is meaningful in mediating affective (anxiety-related behavior) nicotine withdrawal behaviors.

The main conclusion from the above studies is that the depletion of CaMKIV is responsible for the anxiolytic-like effect in animals (Table 2). However, advanced studies about the role of the kinase in fear should be performed since we have limited data on this subject. 

## 7. Memory and Its Impairment—Preclinical Studies

Both CaMKII and CaMKIV are the important nexuses of the memory and learning processes, and their proper functioning is required to remember longer the events or things. Moreover, memory deficits occur more often due to increased life expectancy and accompany mood disorders [190]. Here, we highlight the role of these kinases in normal memory functioning as well as in memory disorders such as Alzheimer’s disease in rodents.

### 7.1. CaMKII

CaMKII phosphorylation is critical for long-term potentiation (LTP) induction in the CA1 region of the hippocampus [191,192,193,194]. However, the site of phosphorylation leads to various outcomes. While autophosphorylation at Thr286 is crucial for LTP induction and its blocking impairs LTP and learning, the prevention of inhibitory phosphorylation at Thr305/306 lowers the threshold for LTP formation and results in less fine-tuned spatial learning and contextual discrimination, as well as in impaired reversal learning in the Morris water maze [195]. Additionally, the CaMKIIα phosphorylation of TARPγ-8 (a component of the AMPA receptor complex) is important for LTP [196]. 

The level of phosphorylated CaMKII depends on age and memory training. Moreover, the kinase amount varies across different brain structures [197]. Contextual fear or passive avoidance learning increases the CaMKIIα autophosphorylation in the hippocampus, but not in the amygdala [198,199]. However, such changes occur only in young mice, while training in old individuals does not improve any parameters [198]. On the other hand, Rodrigues and colleagues showed that fear conditioning leads to the CaMKII increase in the lateral amygdala and the inhibition of the kinase dose-dependently impairs the acquisition, but not the expression, of auditory and contextual fear conditioning [200]. The differences may also be the result of different species, strains, or test procedures. The changes in CaMKII level also depend on whether we take into account naïve or “disease modeled” animals, e.g., a decreased level of CaMKII with disrupted LTP and behavioral performance, was observed in olfactory bulbectomized mice [121]. However, in corticosterone-treated mice, the LTP and synaptic plasticity were enhanced with concomitant CaMKII activation [201].

In CaMKIIα knockout and CaMKIIα-T286 mutant mice (with blocked autophosphorylation at threonine-286), scientists observed impaired spatial memory with a concomitant decrease in LTP formation [161,168,169]. Interestingly, CaMKIIα heterozygous knockout mice exhibited severe working memory deficits in the eight-arm radial maze task and delayed alternation task, while the reference memory assessed by the same tasks remained intact [118]. Moreover, in these mice, c-fos expression after working memory assessment in the eight-arm radial maze was predominantly reduced in the dentate gyrus, CA1, and CA3 regions of hippocampus, amygdala, and medial prefrontal cortex—regions that are essential for working memory functioning [13]. Other results were obtained in investigating the role of kinase in permanent memory. CaMKIIα heterozygous knockout mice showed normal learning and memory 1 ± 3 days after training in the Morris water maze and contextual conditioning tasks with deep memory impairment after 10 ± 50 days [170]. Moreover, in this study, CaMKIIα heterozygous mice had impaired cortical, but not hippocampal, long-term potentiation [170]. 

CaMKIIα depletion in mice is also related to impaired contextual fear memory, manifesting in reduced freezing responses in the fear conditioning task [157,167]. However, some study reports that in CaMKII, mutant mice performed normally in contextual fear conditioning. However, in these mice, the disrupted activity of CaMKII was obtained differentially by introducing aspartate at amino acid 286 [169]. The studies on kinase-dead homozygous CaMKIIα (K42R)-KI mice showed severe impairment in hippocampus-dependent context discrimination in fear memory, whereas amygdala-dependent cued fear memory was partially preserved [202]. This suggests that the activity of CaMKII in various brain structures implicates mice performance in behavioral tests. Moreover, the CaMKIIβ knockout mice showed memory impairments [40]. They had impaired recognition memory evaluated in the novel object recognition test. 

Notably, the CaMKII expression’s location also plays a vital role in memory processes [93,203]. The disruption of CaMKII translation in dendrites causes long-term spatial, emotional, and recognition memory impairment without a negative effect on short-term memory [93]. 

The interesting issue raised concerns about the role of CaMKII in the learning process and separately in memory. In three amygdala-dependent tasks (passive avoidance, contextual, and cued fear conditioning), lack of CaMKIIα autophosphorylation led to learning retardation. However, extended training normalized the behavioral outcomes of both wild-type and CaMKIIα-T286 mutant mice [163]. The normal memory formation must involve other parallel signaling pathways resulting in, e.g., CREB or brain-derived neurotrophic factor (BDNF) phosphorylation [163]. Nevertheless, in the light of studies performed by Frankland and colleagues, it may happen that possible memory impairment would be visible after a longer period [170]. On the other hand, the critical time for CaMKII activity and influence on learning was established using the object recognition test in rats [204]. In the perirhinal cortex, CaMKII was active within over 20 and less than 100 min following learning and then returned to baseline, and therefore its increased activity did not persist throughout the 24 h memory delay [204]. Moreover, Wang and colleagues proved the specific time-dependent role of CaMKII, indicating that the first post-learning week is crucial for consolidating long-term memories in the brain [205]. This suggests that CaMKII is critical for learning processes, especially the consolidation stage, rather than memory itself. 

The CaMKII regulates the transcription of genes essential for memory consolidation via CREB (the upregulation of NGFI-B mRNA expression requires the autophosphorylation of CaMKIIα [206]) as well as glutamate receptors density (involved in excitatory synaptic transmission and mechanisms of learning and memory) [207,208]. 

The decreased level of CaMKII in the frontal cortex and hippocampus occurs in various animal models of Alzheimer’s disease, such as amyloid precursor protein transgenic mice or vascular dementia gerbils [209,210]. Moreover, amyloid β disrupts hippocampal long-term-potentiation via inhibition of activity-dependent CaMKII autophosphorylation and AMPA receptor phosphorylation [211]. The importance of synaptic CaMKII in Aβ-induced reduction of AMPA receptors was proved by changing kinase expression. The CaMKII depletion leads to the reduction of AMPA receptors similar to the effect after of Aβ, whilst kinase overexpression prevents Aβ from decreasing AMPA receptors current density [209]. Moreover, spatial training of Tg2576 mice (Alzheimer’s disease animal model) increases autophosphorylation of CaMKIIα in the hippocampus, ameliorates Alzheimer’s disease-like tau and amyloid pathologies, and rescues recent and remote memory [212]. 

However, CaMKII dysregulation may also modulate the toxicity in Alzheimer’s disease. Amyloid precursors protein and tau protein can be phosphorylated by hyperactive CaMKIIα, leading to Aβ and neurofibrillary tangles formation [209,213,214,215]. The hyperactivation of CaMKIIα occurs in the cell bodies, outside the synapses. In CaMKIIα-expressing neurons, an increased number of neurofibrillary tangles has been observed [214,216,217]; however, such a situation does not always occur [214,217,218]. Moreover, the tau may be phosphorylated by several other kinases at CaMKII sites, making it difficult to detect to what extent the CaMKII is responsible for neurofibrillary tangles formation [219]. In senescence-accelerated mouse prone/8 (SAMP8) displaying age-related cognitive defects and brain amyloidosis at earlier stages of life, the mRNA and protein levels of CaMKIIα in the cerebral cortex and hippocampus is increased, whereas their control littermates SAM resistant/1 (SAMR1) has a decreased level of CaMKII [220]. These contradictory results may be explained precisely by the abnormal CaMKII-mediated hyperphosphorylation of amyloid precursor protein and tau proteins in SAMP8 mice, which are vulnerable to the development of pathological brain changes, whilst in normal-aging mice, the CaMKII functions are normal, and over time, they naturally are diminished. To sum up, it seems that optimal activity of CaMKII is required for proper learning and memory functioning without or minimal negative effect for possible amyloids and neurofibrillary tangles creation. 

CaMKII is crucial for memory-improving properties of rivastigmine, sufiniram, or nefiracetam [120,221,222]. Chronic administration of rivastigmine restores the reduced phosphorylation of CaMKII and long-term potentiation in the hippocampus and improves animals’ performance in the Y-maze task, novel object recognition task, passive avoidance task, and Barnes maze task [222]. Importantly, the procognitive effects of rivastigmine administration did not occur in CaMKII knockout mice, suggesting the kinase’s key role for drug activity [222]. Other research studies also highlight that the increase in CaMKII autophosphorylation positively affects LTP [223] and learning processes [224]. Chronic administration of naringin prevents the AD mice memory decline in passive avoidance task [224]. 

CaMKII also plays a significant role in memory impairment caused by toxins, drugs, and heavy metals. Pb^2+^-exposed rats have decreased CaMKII activity, increased substrate affinity of the enzyme, disrupted LTP, and spatial learning deficits [225,226]. Moreover, the activation of CaMKII in the hippocampus may be crucial for reverse in negative morphine effects on spatial memory consolidation [227].

Taken together, the CaMKII is a rudimentary kinase for learning and memory processes. In knockout animals, we observe disrupted emotional, recognition, spatial, and working memory (Table 2). During learning and LTP formation, the expression of CaMKII increases, which is also observed after the administration of memory-enhancing compounds.

### 7.2. CaMKIV 

CaMKIV plays a significant role in memory consolidation processes and well as long-term potentiation. CaMKIV knockout mice have disrupted synaptic plasticity on many levels [166]. They have impaired long-term potentiation formation in CA1 neurons of the hippocampus, exhibit a lack of late-phase long-term depression (LTD) in cerebellar Purkinje cells, and impaired CREB activation [166]. Notably, LTP phases are not equally affected by CaMKIV malfunctioning. Inhibition of kinase activation impairs late LTP (L-LTP) with normal basic synaptic function and early LTP (E-LTP) formation [113]. It clearly shows that the short-term memory and acquisition phase is not affected, while both consolidation and retrieval phases in the long-term memory are disrupted. 

Interestingly, different results were reported regarding the function of CaMKIV in memory-assessing behavioral tasks. Genetic deletion of CaMKIV did not reflect on animals’ performance in spatial memory tests (Morris water maze, Barnes maze, eight-arm radial maze) [153,166], while transgenic mice expressing a dominant-negative CaMKIV achieved worse results in the Morris water maze than the wild-type animals and used cue–platform association strategy instead of spatial strategy [113]. The possible explanations concern the fact that some LTP forms may have occurred despite the mutation and be sufficient for proper spatial learning or CREB, an important memory factor, can be phosphorylated in other molecular pathways.

Notably, CaMKIV controls not only spatial but also emotional memory. Both genetic depletion of CaMKIV and transgenic kinase inhibition lead to learning deficits in fear conditioning [113,153,162]. The lack of behavioral effect of CaMKIV knockout mice in spatial tasks, but not fear-related tasks, shows that different CREB activation pathways preferentially encode different forms of memory and that CaMKIV deficiency may be compensated by other neural mechanisms. Interestingly, even though both fear conditioning and passive avoidance investigate emotional memory, studies report ambiguous results. Even if CaMKIV knockout mice display impairment in the fear condition, they cope with passive avoidance task similar to their wild-type littermates [153,158]. However, in the modified version (temporal dissociative passive avoidance task), CaMKIV depletion was linked to animals’ worse performance [158], which suggests that training intensity and complexity requires well-functioning CaMKIV. 

The long-term memory impairments are also seen in CaMKIV knockout mice performing eyeblink conditioning test [164] or during vestibulo-ocular reflex testing [165], which assesses cerebellar-dependent motor memory.

On the other hand, the overexpression of CaMKIV in the forebrain promotes social and fear long-term memory formation [112]. Looking deeper, the LTP, but not basal synaptic transmission, is significantly enhanced in CaMKIV mice and prevents the age-related decrease in kinase level and disruption of the memory formation [112]. Studies suggest that CaMKIV promotes early synaptic potentiation by activating new protein synthesis in the anterior cingulate cortex and thus fear memory improvement [171,228]. Additionally, the increased expression of CaMKIV enhances 4–7.5 Hz oscillations (involved in attention and learning) during trace fear conditioning and slow delta oscillations during NREM sleep (involved in memory consolidation) [172]. 

CaMKIV is an essential factor in the development of many cognitive deficits caused either by toxins, drugs, or age. The arsenic-induced decrease in CaMKIV expression subsequentially repressed cerebellar LTD formation, manifested by worse performance of mice in spatial memory task [229,230]. The memory disturbances in the rat model of Alzheimer’s disease caused by Aß peptide infusion are also related to a decrease in CaMKIV level and its downstream targets such as CREB [231]. Moreover, in the pathophysiology of Alzheimer’s disease, CaMKIV is one of the most significant factors changed by disease development [232]. Scientists noticed that intracellular accumulation of hTau causes synapse and memory deficits through CaN-mediated dephosphorylation/inactivation of CaMKIV/CREB signaling in the nuclei [232]. Interestingly, as in APP23 mice (model of Alzheimer’s disease), we can observe learning and memory deficits in NCKX2 (K^+^-dependent Na^+^/Ca^2+^ exchanger) heterozygous mice, as well as attenuated hippocampal LTP with reduced CaMKII and CaMKIV activity [233]. Moreover, rivastigmine normalizes the decreased level of phosphorylated CaMKIV, but this kinase is not essential for memory-improving properties [222]. However, other studies have shown that CaMKIV may have a negative effect on Alzheimer’s disease development. This kinase may excessively phosphorylate the tau protein and consequently cause neurofibrillary tangles formation [234,235]. In rats, the regulation of CaMKIV activity by genistein displays a beneficial effect in Alzheimer’s disease prevention both on the behavioral (improved spatial learning and memory) and cellular levels (reduced hippocampal neuron damage and level of p-tau) [235]. Nevertheless, the constitutive activation of CaMKIV and CREB is mostly observed in familial Alzheimer’s disease [236]. 

To conclude, CaMKIV as CaMKII is essential for LTP formation and memory functioning. CaMKIV knockout mice have spatial, emotional, and motor deficits (Table 2). In rodents with induced memory disorders, we primarily observe the decrease in the kinase level. Considering that the activation of CaMKIV is beneficial for proper memory functioning, procognitive agents should normalize the disrupted CaMIV expression.

## 8. Conclusions

The CaMKII and CaMKIV are essential kinases controlling mood and memory functions. Their dysfunction underlies various neurodegenerative and neuropsychiatric disorders. Moreover, both CaMKII and CaMKIV mediate the therapeutic effects of many central-acting drugs on the molecular level. On the basis of the reviewed literature, in order to achieve an antidepressant-like and procognitive effect, one finds that compounds should generally increase the phosphorylation of CaMKII and CaMKIV (Table 3). On the other hand, most studies suggest that anxiolytic-like activity results from kinases inhibition. Unfortunately, the data on the role of CaMKII and CaMKIV in anxiety are still limited. 

The scientific findings show that the alterations in CaMKII and CaMKIV functioning are not responsible for all symptoms observed in depression, anxiety, or memory impairments. However, considering the critical role of CaMKII and CaMKIV in glutamatergic neurotransmission, which underlies many neuropsychiatric and neurodegenerative disorders, drugs targeting these kinases might help modulate some disease symptoms. Therefore, CaMKII and CaMKIV may be attractive targets for novel drugs and should be considered when designing new compounds.

## Figures and Tables

**Table 1:** Comparison of calcium/calmodulin-dependent kinases (CaMK) II and IV, considering brain regions, cell types, functions, and disease-like states in rodents associated with dysfunctions of kinases.

	CaMKII	CaMKIV
**Brain regions**	prefrontal cortex [10,11], hippocampus [12,13], amygdala [14], lateral habenula [15,16]	hippocampus [17], nucleus accumbens [18]
**Cell types**	isoforms α and β: brain neurons; isoforms γ and δ: present in every examined tissue [19,20]	nervous tissue (mostly in cerebellum’s granular cells [21]), thymus (especially T-lymphocytes), spleen, and testis [22]
**Function**	regulation of various cellular functions by phosphorylating a wide range of substrates, e.g., regulation of immune system [23,24,25], cardiac function [26,27,28], pain [29]	regulation of various cellular functions by phosphorylating a wide range of substrates, e.g., controlling transcription factors such as CREB, ATF-1, SRF [30,31,32,33], blood pressure [34], immune system [35,36,37], microtubule dynamics [38]
**Examples of dysfunction and associated disease-like state in rodents**	abnormal phosphorylation—Angelman syndrome [39], β isoform overexpression—depressive-like symptoms [15], β isoform knockout—anxiety-related behavior [40], inhibition of α isoform expression—anxiolytic-like effect [41]	overexpression and increased activity—various types of cancer [42], knockout or inhibition—depressive-like state [17,43]

**Table 2:** Summary of the effects of calcium/calmodulin-dependent kinases II and IV genetic manipulation in rodents.

	CaMKII				CaMKIV		
Genetic Manipulation	Behavioral Assay	Effect in Rodents	Reference	Genetic Manipulation	Behavioral Assay	Effect in Rodents	Reference
Homozygous CaMKIIα knockout	FST	no changes	[116]	CaMKIV knockout	FST, TST	no changes	[153]
Heterozygous CaMKIIα knockout	FST	depressive-like state	[118]	homozygous CaMKIV knockout	FST, TST	depressive-like state	[154]
CaMKIIβ overexpression	FST, SPT	antidepressant-like effect	[15,119]	-	-	-	-
CaMKIIβ knockdown (hippocampus) in UCMS rats	SPT, FST	antidepressant-like effect	[119]	-	-	-	-
CaMKIIα knockdown	OFT	anxiolytic-like effect	[155]	homozygous CaMKIV knockout	EPM, LDT, acoustic startle and prepulse inhibition	anxiolytic-like effect	[153,156]
Hetero- and homozygous CaMKIIα knockout	FC	anxiolytic-like effect	[157]	homozygous CaMKIV knockout	marble-burying test, light dark box	anxiolytic-like effect	[158]
Hetero- and homozygous CaMKIIβ knockout	EPM, OFT	anxiolytic-like effect	[40]	hetero- and homozygous CaMKIV knockout + nicotine dependence and withdrawal	conditioned place preference test	anxiolytic-like effect (during withdrawal)	[159]
CaMKIIα overexpression	OFT, EZM, RT	anxiety-like state	[160]				
CaMKIIα-T286 mutant mice	Morris water maze	memory impairment	[161]	homozygous CaMKIV knockout	FC		[158,162]
CaMKIIα-T286 mutant mice	PA, cued FC	memory impairment	[163]	homozygous CaMKIV knockout	eyeblink conditioning test, vestibulo-ocular reflex testing		[164,165]
Heterozygous CaMKIIα knockout	RAM, delayed alternation task	working memory impairment	[13,118]	homozygous CaMKIV knockout	Morris water maze, Barnes maze, RAM		[166]
Heterozygous CaMKIIα knockout	FC	memory impairment	[157,167]	CaMKIV knockout	Barnes maze, RAM, PA	no changes	[153]
Heterozygous CaMKIIα knockout	Morris water maze	memory impairment	[168]	CaMKIV knockout	FC	memory impairment	[153]
CaMKII-Asp-286	Barnes maze, FC	spatial memory impairnent, no effect on contextual memory	[169]	heterozygous CaMKIVα knockout	Morris water maze, FC	memory impairment	[113]
Heterozygous CaMKIIα knockout	Morris water maze, FC	long-term memory impairment	[170]	CaMKIV overexpression	FC	memory enhacement	[171,172]
Kinase-dead CaMKIIα (K42R)-knockin	Morris water maze, FC	spatial and contextual memory impairment		CaMKIV overexpression	PA, social recognition test		[112]
Hetero- and homozygous CaMKIIβ knockout	NOR	memory impairment	[40]				

CaMKII—calcium/calmodulin-dependent kinase II, CaMKIV—calcium/calmodulin-dependent kinase IV, EPM—elevated plus maze test, EZM—elevated zero maze, FC—fear conditioning, FST—forced swim test, LDT—light/dark test, NOR—novel object recognition task, OFT—open field test, PA—passive avoidance, RAM—radial arm maze, RT—rotarod test, SPT—sucrose preference test, TST—tail suspension test, UCMS – unpredictable chronic mild stress.

**Table 3:** Potential modulators of CaMKII and/or CaMKIV and their activity in animal tests/models of neuropsychiatric and neurodegenerative disorders.

Potential Modulator	Action	Animal Models/Tests	Reference
**Antidepressant-Like Activity**			
			
KN-62	- inhibition of CaMKII - inhibition of GluA1 (Ser831) phosphorylation	FST, SPT, and ethanol withdrawal (in rats)	[126,141,142,143,144,146]
			
Paroxetine, venlafaxine, fluvoxamine (long treatment)	- activation of CaMKII in synaptic vesicles and synaptic cytosol - activation of CaMKIIα phosphorylation in hippocampus - activation of synaptotagmin phosphorylation	-	[127,130,131,132]
			
Fluoxetine (chronic)	- inhibition of CaMKII expression in nucleus accumbens - inhibition of ΔFosB binding to the CaMKIIα promoter - inhibition of lysine-9 histone H3 acetylation and activation its dimethylation at the CaMKIIα promoter in nucleus accumbens	chronic social defeat stress and SPT (in mice)	[128]
			
Fluoxetine, desipramine, Reboxetine (long treatment)	- inhibition of CaMKII - activation of Thr(286) phosphorylation	-	[129]
			
Imipramine (chronic)	- inhibition of CaMKIIβ (in lateral habenula or hippocampal soluble fraction) - inhibition of GluR1	congenitally learned helplessness (in rats)	[15,134]
			
Fluoxetine (chronic)	- inhibition of CaMKIIβ in hippocampus CA1 - inhibition of the p38 MAPK phosphorylation - inhibition of transcription factor 2 activating	FST and SPT during chronic unpredictable mild stress and LPS-induced model of depression (in rats)	[119]
			
Fluvoxamine, desipramine (chronic)	- activation of CaMKII in presynaptic vesicles of prefrontal /frontal cortex - activation of synaptotagmin phosphorylation	-	[10]
			
Desmethylimipramine (chronic)	- activation of CaMKII in hippocampal synaptic vesicle fraction	-	[133]
			
Ketamine	- activation of CaMKII - activation of GluA1 in the hippocampus	FST, TST, and NSFT (in mice)	[135]
			
Ketamine	- inhibition of CaMKIIα overexpression in hippocampus - inhibition of GluN2B phosphorylation in the hippocampus	OFT, FST, and NSFT during LPS-model of depression (in mice)	[136]
			
Sunifiram (chronic)	- activation of CaMKII phosphorylation in OB mice in the hippocampus CA1 - activation of glycine-bindind site of NMDAR	no effect in TST (in OB mice)	[120]
			
Nefiracetam (chronic)	- activation of CaMKII and CaMKIV phosphorylation in OB mice in the hippocampus CA1 as well as in amygdala and prefrontal cortex - activation of CREB phosphorylation in amygdala and prefrontal cortex	TST and FST (in OB mice)	[137]
			
SAK3 (ethyl 8’-methyl-2’,4-dioxo-2-(piperidin-1-yl)-2’H-spiro[cyclopentane-1,3’-imidazo[1,2-a]pyridine]-2-ene-3-carboxylate) (chronic)	- activation of CaMKII and CaMKIV phosphorylation in OB mice - activation of T-type calcium channel	TST, FST, and SPT (in OB mice)	[138]
			
Guanxin Danshen formula	- inhibition of CaMKII	FST, TST, and SPT during unpredictable chronic mild stress (in rats)	[139]
			
3,5,6,7,8,3,4′-Heptamethoxyflavone	- activation of p-CaMKII in the hippocampus	FST and TST during corticosterone-induced depression (in mice)	[140]
			
Baicalin	- inhibition of CaMKII	FST, TST, and SPT during mice chronic unpredictable stress (in mice)	[150]
			
Ursolid acid, creatine, ferulic acid, (octylseleno)-xylofuranoside, memantine and 7-fluoro-1,3-diphenylisoquinoline-1-amine	- activation of CaMKII	FST or TST (in mice)	[141,142,143,144,145,146,147,148,149]
			
Nicotine	- activation of CaMKIV - activation of α7-nAChRs	TST and FST (in CaMKIV knockout mice)	[43]
			
Fluoxetine (chronic)	- activation of CaMKIV	SPT and OFT during chronic unpredictable stress (in rats)	[174]

**Anxiolytic-like activity**			
			
TatCN21peptide	- inhibition of CaMKII and αCaMKII - inhibition of NR2B phosphorylation	EPM and OFT (in rats)	[41]
			
Diazepam	- activation of p-CaMKII in the hippocampus and striatum - inhibition of MEK and ERK1/2	OFT and EPM (in mice)	[187]
			
Memantine	- inhibition of CaMKII phosphorylation - inhibition of ERK signalling	OFT and EPM during chronic alcohol exposure (in rats)	[188]
			
Genistein	- activation of p-CaMKII - activation of the serotonergic transmission in the amygdala	EPM and contextual freezing behavior during posttraumatic stress disorder (in rats)	[189]

**Procognitive activity**			
			
Rivastigmine (chronic)	- activation of CaMKII and CaMKIV phosphorylation - activation of long-term potentiation in the hippocampus	Y-maze task, NOR, passive avoidance task, and Barnes maze task (in OB mice)	[222]
			
Sunifiram	- activation of CaMKII - activation of the glycine-binding site of NMDAR	Y-maze task, NOR (in OB mice)	[120]
			
Nefiracetam	- activation of CaMKII - activation of GluA1 phosphorylation - activation of long-term potentiation in rats	-	[221]
			
Naringin (chronic)	- activation of CaMKII phosphorylation - activation of AMPA phosphorylation - activation of long-term potentiation in mice model of Alzheimer’s disease	-	[224]
			
Genistein	- inhibition of CaMKIV in the hippocampus - inhibition of tau phosphorylation	special learning and memory in Morris water maze during Alzheimer ‘s disease model (in rats)	[235]

CaMKII—calcium/calmodulin-dependent kinase II, CaMKIV—calcium/calmodulin-dependent kinase IV, EPM—elevated plus maze test, FST—forced swim test, LPS—lipopolysaccharide, NOR—novel object recognition task, NSFT—novelty suppressed feeding test, OB mice—olfactory bulbectomized mice, OFT—open field test, p-CaMKII—phosphorylated-CaMKII, SPT—sucrose preference test, TST—tail suspension test.

## Data Availability

Not applicable.
